# Deciphering Early and Progressive Molecular Signatures in Alzheimer’s Disease through Integrated Longitudinal Proteomic and Pathway Analysis in a Rodent Model

**DOI:** 10.3390/ijms25126469

**Published:** 2024-06-12

**Authors:** Hamad Yadikar, Mubeen A. Ansari, Mohamed Abu-Farha, Shibu Joseph, Betty T. Thomas, Fahd Al-Mulla

**Affiliations:** 1 Department of Biological Sciences, Faculty of Science, Kuwait University, Sabah AlSalem University City, Kuwait City 13060, Kuwait; 2 OMICS Research Unit, Research Core Facility, Faculty of Medicine, Kuwait University, Kuwait City 13110, Kuwait; bety.tomas@ku.edu.kw; 3 Department of Pharmacology and Toxicology, Faculty of Medicine, Kuwait University, Kuwait City 13110, Kuwait; mubeen.ansari@ku.edu.kw; 4 Department of Translational Research, Dasman Diabetes Institute, Kuwait City 15462, Kuwait; mohamed.abufarha@dasmaninstitute.org (M.A.-F.); fahd.almulla@dasmaninstitute.org (F.A.-M.); 5 Department of Special Service Facility, Dasman Diabetes Institute, Kuwait City 15462, Kuwait; shibu.joseph@dasmaninstitute.org

**Keywords:** intracerebroventricular streptozotocin, Alzheimer’s disease proteomics, temporal expression profiling, neurodegenerative biomarkers, pathway dysregulation

## Abstract

Alzheimer’s disease (AD), the leading cause of dementia worldwide, remains a challenge due to its complex origin and degenerative character. The need for accurate biomarkers and treatment targets hinders early identification and intervention. To fill this gap, we used a novel longitudinal proteome methodology to examine the temporal development of molecular alterations in the cortex of an intracerebroventricular streptozotocin (ICV-STZ)-induced AD mouse model for disease initiation and progression at one, three-, and six-weeks post-treatment. Week 1 revealed metabolic protein downregulation, such as Aldoa and Pgk1. Week 3 showed increased Synapsin-1, and week 6 showed cytoskeletal protein alterations like Vimentin. The biological pathways, upstream regulators, and functional effects of proteome alterations were dissected using advanced bioinformatics methods, including Ingenuity Pathway Analysis (IPA) and machine learning algorithms. We identified Mitochondrial Dysfunction, Synaptic Vesicle Pathway, and Neuroinflammation Signaling as disease-causing pathways. Huntington’s Disease Signaling and Synaptogenesis Signaling were stimulated while Glutamate Receptor and Calcium Signaling were repressed. IPA also found molecular connections between PPARGC1B and AGT, which are involved in myelination and possible neoplastic processes, and MTOR and AR, which imply mechanistic involvements beyond neurodegeneration. These results help us comprehend AD’s molecular foundation and demonstrate the promise of focused proteomic techniques to uncover new biomarkers and therapeutic targets for AD, enabling personalized medicine.

## 1. Introduction

Alzheimer’s disease (AD), the leading cause of dementia globally, presents a constellation of neurodegenerative manifestations characterized by cognitive decline and memory loss [1]. The disease’s insidious onset and complex etiology, compounded by the absence of curative treatments, underline the urgency for advanced research into its pathophysiological foundations. The increasing worldwide occurrence of AD dictates a more profound comprehension as well as innovative strategies for diagnosis and treatment because of its significant impact on millions of people and their families [2].

Predominantly, AD is characterized by amyloid-beta (Aβ) plaque deposition and tau protein hyperphosphorylation [3], yet these hallmarks do not entirely account for the disease’s multifactorial nature [4]. Moreover, current biomarkers lack sensitivity and specificity, such as Aβ and tau levels in cerebrospinal fluid (CSF) and imaging techniques. One of the significant challenges in AD research is the heterogeneity of the disease, which complicates the identification of universal biomarkers and therapeutic targets [5]. Recent paradigms have shifted focus toward metabolic dysfunction, implicating impaired glucose metabolism and insulin resistance in the brain as pivotal factors in AD’s early stages [6,7,8,9]. A critical gap slows down the pressing need for early detection and intervention in AD in our understanding and identification of precise biomarkers and effective therapeutic targets. This metabolic perspective of AD pathogenesis has engendered the development of novel animal models that more closely replicate the human condition [10]. One such model, involving the intracerebroventricular administration of streptozotocin (ICV-STZ), has been seminal in emulating AD’s metabolic and cognitive disturbances [11]. The ICV-STZ-induced rodent model has emerged as a valuable tool for replicating the disease’s metabolic dysfunction, including impaired glucose metabolism and insulin resistance within the brain [12,13,14]. The ICV-STZ model reflects these metabolic irregularities and replicates the cognitive impairments characteristic of AD, making it an instrumental framework for investigating the disease [11,15,16].

Our AD study hypothesis suggests that metabolic dysfunction, particularly poor glucose metabolism and insulin resistance in the brain, is critical in the disease’s initial phases [17,18]. This metabolic concept is vital for connecting the biochemical pathways to the cognitive decline symptoms seen in AD and guiding the experimental investigations that shape our methodological emphasis. Our study focuses on how changes in protein expression and enzyme activity provide early biomarkers for AD before the typical signs like Aβ buildup and tau are hyperphosphorylated. We aim to create a dynamic disease progression model by systematically outlining molecular changes using advanced proteomic tools, combining metabolic dysfunction with synaptic and cytoskeletal degradation.

To test our hypothesis, we employed advanced proteomic technologies that heralded a new neurodegenerative disease research era. Mass spectrometry (MS)-based proteomics offers a powerful lens through which the molecular mechanism of diseases like AD can be examined in unprecedented detail [19,20,21]. This approach allows for the comprehensive profiling of the proteome, providing insights into the dynamic changes in protein expression and interactions that occur as the disease progresses. By mapping these proteomic landscapes, researchers can identify critical proteins and pathways that play pivotal roles in the onset and development of AD, offering potential avenues for discovering novel biomarkers and therapeutic targets [22].

This study presents a longitudinal proteomic analysis to explore the temporal evolution of molecular changes in the ICV-STZ-induced AD rodent model. By employing data-independent acquisition (DIA) and data-dependent acquisition (DDA) MS techniques, we aimed to examine the cortical proteome at multiple post-treatment intervals comprehensively. The dual MS strategy enables an in-depth proteomic analysis, uncovering significant alterations in protein expression and perturbed pathways critical for understanding AD’s pathogenesis. To this end, our study harnesses the power of Ingenuity Pathway Analysis (IPA) to explore the proteomic alterations identified in the ICV-STZ-induced AD rodent model. IPA’s advanced computational tools and machine learning algorithms allow for predicting the functional consequences of the observed proteomic changes, offering insights into the roles of specific proteins and pathways in the disease’s pathology. By mapping these molecular alterations to known networks of gene and protein interactions, we can untangle the complex web of biological processes that underlie AD’s progression. This integrative analysis promises to highlight critical pathways that are dysregulated in the disease, providing fertile ground for identifying biomarkers and therapeutic targets.

## 2. Results

### 2.1. Overview of Identified Proteins and Peptides in STZ and Control Groups Reveals Significant Overlaps

**Figure 1:**
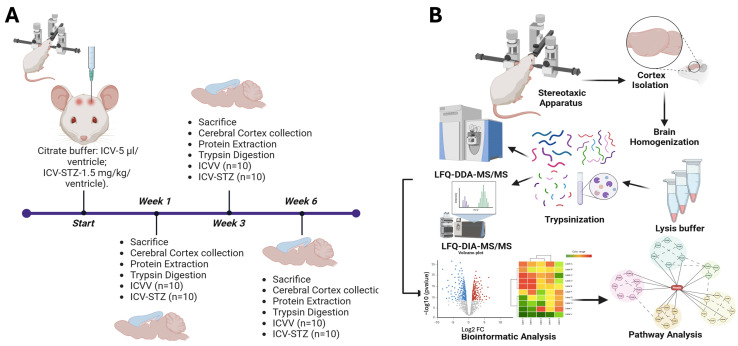
Schematic overview of the experimental workflow for the longitudinal proteomic analysis in an Alzheimer’s disease rodent model. (**A**) Schematic timeline of the experimental setup. Rats were divided into two groups: one receiving intracerebroventricular (ICV) administration of vehicle (ICVV) and the other receiving streptozotocin (ICV-STZ) to induce Alzheimer ’s-like pathology. The timeline includes three key time points: the start, week 1, week 3, and week 6 post-administration. At each time point, rats were sacrificed, and prefrontal cortex (PFC) tissues were collected. Each group consisted of 10 rats (n = 10 for ICVV and ICV-STZ). (**B**) workflow of sample processing and analysis. Post-cortex isolation using a stereotaxic apparatus, tissues were homogenized and lysed. The proteins were then digested into peptides through trypsinization. Peptides underwent cleanup using C_18_ columns and were concentrated. The prepared samples were analyzed using two mass spectrometry techniques: label-free quantification data-dependent acquisition (LFQ-DDA-MS/MS) for broad proteomic profiling and label-free data-independent acquisition (LFQ-DIA-MS/MS) for targeted quantification. The bioinformatics analysis included generating volcano plots to identify significantly altered proteins, hierarchical clustering for pattern recognition, and pathway analysis to elucidate the molecular mechanisms underlying observed proteomic changes. This approach facilitates the identification of temporal proteomic alterations and the discovery of potential biomarkers and therapeutic targets for Alzheimer’s disease.

In our longitudinal proteomic analysis of an Alzheimer’s disease (AD) rodent model, we followed the protein expression dynamics at various stages of disease progression using a robust experimental design (Figure 1). The subjects were divided into two cohorts, intracerebroventricularly (ICV) administered with either the vehicle (ICVV) or streptozotocin (ICV-STZ), the latter inducing AD-like neuropathology. Progression of proteomic changes were tracked at weeks 1, 3, and 6 (1 W, 3 W, 6 W) post-administrations. Rigorous sample collection from cortex tissues was ensured by stereotaxic isolation, succeeded by a series of preparatory steps including homogenization, lysis, and centrifugation, culminating in protein digestion into peptides by trypsinization. We subsequently refined the peptides using C_18_ columns and concentrated them through speed vacuum concentration, equipping us with high-resolution mass spectrometric analysis. The proteomic analysis was two-pronged: employing label-free quantification data-dependent acquisition (LFQ-DDA-MS/MS) for a sweeping proteomic profile alongside label-free data-independent acquisition (LFQ-DIA-MS/MS) for fine-tuned quantification. This dual approach allowed us to juxtapose peptide abundance across the delineated temporal milestones, bridging the quantitative divide in proteomic alterations.

In the proteomic analysis of ICV-STZ-treated AD rodent models, proteins were selected based on criteria ensuring high confidence and biological relevance (Table 1). We included proteins with 15 or more peptide spectrum matches (PSMs) and at least 4 unique peptides to ensure robust protein identification and quantification. Only proteins that demonstrated statistically significant abundance changes were considered (adj. *p*-value < 0.05). To focus on the most biologically significant alterations, proteins with log_2_ abundance ratios of 2.00 or more and those less than or equal to −2.00 were included, highlighting proteins with at least a 4-fold change in expression levels. These stringent selection parameters ensured that the data presented captured the most prominent and reliable protein expression changes relevant to the AD pathology induced by ICV-STZ treatment (Table 1). The complete proteins and peptides identified in the ICV-STZ and control groups within the cortex datasets are detailed in Table S1. Detailed protein identification metrics and associated statistical analyses are provided in Figure S1, offering a comprehensive overview of the proteomic landscape at each time point post-treatment.

### 2.2. Identification of Differentially Abundant Proteins Offers Insight into Early AD Neuropathophysiology

**Figure 2:**
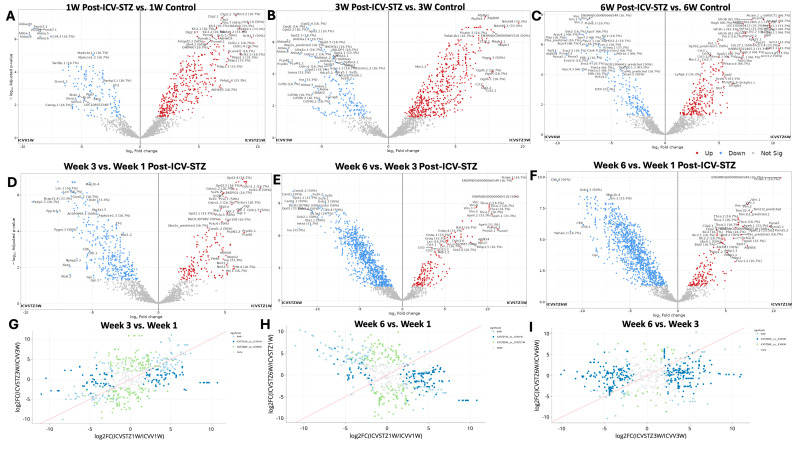
Differential proteomic profiling across time points in an ICV-STZ AD rodent model. Panels (**A**–**C**) represent volcano plots displaying the log2 fold change of protein expressions in the cortex of rats induced with Alzheimer’s pathology using intracerebroventricular streptozotocin (ICV-STZ) at weeks 1, 3, and 6, respectively, against their corresponding controls (ICVV). The x-axis shows log2 fold change between groups, and the y-axis represents the negative log10 of the adjusted *p*-value, indicating the significance of the change. Red dots indicate proteins with a statistically significant increase in expression, blue dots represent a significant decrease, and gray dots denote proteins with no significant change. Panels (**D**–**F**) juxtapose the same time points (weeks 1, 3, and 6 post-ICV-STZ administration) directly against each other to highlight the temporal progression of proteomic alterations. Panels (**G**–**I**) are scatter plots comparing the log2 fold changes between two time points, illustrating the correlation of proteomic changes for the disease model. In all scatter plots, the red line indicates the unity line, where equal expression changes between the time points would lie. Green dots above the line indicate a more significant fold change at the later time point (e.g., week 3 vs. week 1), while blue dots below the line indicate a more significant fold change at the earlier time point. Each dot in the volcano plot is annotated with the protein identifier and the fold change value where space allows. Proteins were considered significantly altered with a fold change threshold set at two and an adjusted *p*-value of less than 0.05.

Volcano plots (Figure 2A–F) represent the comparative proteomic landscape, where each point denotes an individual protein’s relative abundance, contrasted between ICV-STZ and ICV-Vehicle for the three time points. Red points highlight proteins with increased abundance (upregulated), blue points denote decreased abundance (downregulated), and grey points indicate proteins without significant changes.

One-week post-administration (Figure 2A), out of 1041 proteins analyzed, 432 exhibited exclusive significant differential abundance. The 1 W time point shows a significant downregulation for proteins such as Aldoa, Psmb7.1, and Aldoart3, Cct4.2 (FC < −5; adj. *p*-values < 0.05). In contrast, Clip2, Sptbn2, Pdap2, Dclk1, Ndufa9 emerge as significantly upregulated (FC > 7; adjusted *p*-values < 0.05). At the 3 W juncture, proteins such as Gdpd2 and Ube2o are significantly downregulated (Figure 2B). Dclk1 and Ndufa proteins show a consistent increase in abundance, illustrated by their red-colored point. At 6 W, we observed proteins like Prdx6, Bat2, Xpo7, and Vim identified as having significant downregulation (FC < −7.5; adjusted *p*-values < 0.0001). They stand out in the blue zone, indicating a significant increase in protein abundance. Conversely, proteins such as Alcam, Idh3B, Slc1a4, Arhgap1, and Cplx2 are significantly upregulated (Figure 2C).

We compared the proteomic landscape across different time points post-ICV-STZ administration to discern the evolution of protein regulation patterns pertinent to AD-like pathology (Figure 2D–F). The transition from 1 W to 3 W reveals a notable downregulation in proteins such as Map1b.4, Lxn.1, Pebp (FC < −5; adjusted *p*-values < 0.0001). These early alterations in abundance reflect initial cellular responses to the induced pathological state. On the opposite spectrum, proteins such as Gpd2.4, Gstm1.1, Dclk1.6, and Sv2b display a significant upregulation (FC > 5). As we move further along the timeline, examining the proteomic changes from 3 W to 6 W, we observe proteins such as Cand1.2, Gpd1, and Sv2b remain prominently downregulated, and proteins such as Vim, Prdx6, and Tbca are upregulated, supporting the notion of sustained biological responses or further advancement of disease mechanisms.

**Figure 3:**
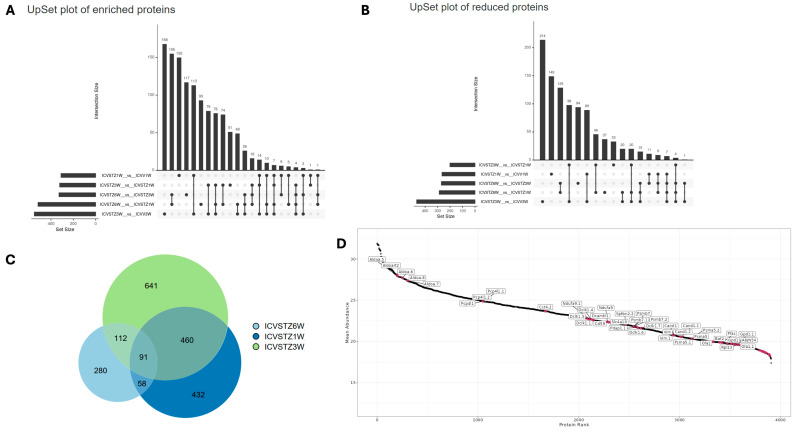
Dynamic Proteomic Patterns in a Streptozotocin-Induced Alzheimer’s Disease Rodent Model Over Time. Panel (**A**) displays an UpSet plot for proteins with enriched abundance, indicating the number and intersections of proteins upregulated at weeks 1, 3, and 6 post-ICV-STZ administration. Panel (**B**) shows an UpSet plot for proteins with reduced abundance, highlighting the count and intersection points of downregulated proteins at the corresponding time points. Panel (**C**) presents a Venn diagram detailing the overlap and unique counts of significantly altered proteins at each time point, illustrating the study’s progression and changes in protein expression. Panel (**D**) demonstrates a protein rank abundance plot, ranking proteins by their abundance in the study and annotating those of particular interest.

Proteins significantly upregulated in 6 W compared to 1 W are shown in red and predominantly in the right upper quadrant of the plot (Figure 2F). This cluster of upregulated proteins, such as Vim (Vimentin), Prdx6 (Peroxiredoxin-6), and Tbca (Tubulin folding cofactor A), indicates a substantial increase in their abundance, signifying a response to prolonged STZ treatment. Conversely, downregulation in 6 W compared to 1 W included Cltb (Clathrin light chain B) and Gnb2 (Guanine nucleotide-binding protein G(I)/G(S)/G(T) subunit beta-2). The correlation of fold changes in protein abundance between the different time points provides a visual semblance of the protein expression stability or fluctuation as the disease pathology evolves (Figure 2G–I). A bi-dimensional correlation between 1 W and 3 W (Figure 2G), 3 W and 6 W (Figure 2H), and 1 W and 6 W (Figure 2I) explains the longitudinal proteomic flux. Here, the grey points elucidate proteins that did not meet the significance threshold across the comparative weeks. This might indicate a homeostatic proteomic equilibrium or a transitional state in the disease’s trajectory. A pattern emerges where a cluster of proteins in the light blue points suggests a group of proteins consistently upregulated from the first to the third week, which may indicate progressive disease pathology or a heightened cellular response to the initial STZ insult.

A significant shift between 1 W and 6 W towards the blue area (lower right quadrant) can be observed (Figure 2H). These proteins are more abundant at 1 W and show a relative decrease by 6 W, suggesting a potential reversal or downregulation in the latter stages of the observation period. Proteins upregulated (light green) from 1 W to 6 W and remaining upregulated by W6 may play critical roles in the chronic phases of the STZ-induced AD-like pathology. The trajectory of proteomes from W3 to W6 is compared directly (Figure 2I). A spread of dark blue dots in the left and right halves of the plot indicates proteins that had increased by 3 W but then decreased in abundance by 6 W, possibly suggesting transient responses or adaptations that may not be sustained in the longer term. Conversely, the light blue dots indicate proteins whose abundance continues to rise, which could be involved in ongoing disease processes or represent a delayed response to the STZ treatment. Principal Component Analysis (PCA) of the proteomic profiles and their distribution across all experimental groups is depicted in Figure S2. Figure S3 illustrates the distribution of peptide intensities, an additional validation layer for our proteomic quantification approach.

### 2.3. Temporal and Comparative Proteomic Profiling Reveals Differential Cortex Protein Dynamics in ICV-STZ Treated Rats

The UpSet plot illustrates the intricate landscape of enriched protein expression across different experimental conditions (Figure 3A,B). The horizontal bars indicate the number of proteins uniquely identified in each set. In contrast, the vertical bars represent the size of the intersection, showing the number of proteins common between the sets. The upregulated protein plot (Figure 3A) indicates a prominent intersection involving over 150 proteins shared between the 6 W vs. 3 W and 6 W vs. 1 W conditions. In contrast, the downregulated protein plot (Figure 2B) presents a peak intersection with 129 proteins between 6 W vs. 3 W and 6 W vs. 1 W comparison.

The uniform distribution seen in the enrichment plot, with intersection counts such as 155 and 117 for other sets, contrasts with the reduced plot, where there is a significant decrease following the most prominent intersection, descending to counts of 149 and 129. This indicates a more variable pattern of protein downregulation. The individual set sizes differ significantly, with the reduction plot (Figure 3B) exhibiting a broader range of unique proteins, especially in the 300–400 set size range for 3 W vs. control, compared to the upregulated plot (Figure 3A). The temporal aspect reveals a shift; while the shared downregulated proteins diminish over time, the shared upregulated proteins amplify, as evidenced by the intersection size growing from 6 W to 1 W comparisons (Figure 3A).

The Venn diagram represents the unique and shared proteins identified at different time points following the ICV-STZ treatment model (Figure 3C). The chart shows 641 unique proteins in the 6 W group, indicating a substantial proteomic alteration occurring 6 W post-treatment. The 1 W group shows 280 unique proteins and the 3 W group presents 432 unique proteins, highlighting the temporal dynamics of the proteome response.

The intersection of all three groups, where 91 proteins are shared, suggests a core set of proteins consistently affected at all examined time points, which may be central to the disease’s pathology or the model’s response to the treatment. Notably, the most significant overlap between the 3 W and 6 W groups, with 460 shared proteins, suggests that the proteomic changes initiated at 3 W persist or evolve up to 6 W. In contrast, the intersection between the 1 W and 6 W groups is comparatively more minor, with 58 proteins, implying a more significant proteomic shift occurring after the 1 W and before the 6 W. Protein rank plot with proteins labeled with higher abundance is highlighted in (Figure 3D). Proteins from the Aldoa family are found in the top left, indicating a high abundance within the sample set. As we move to the right, there is a gradual transition to proteins such as Vim, Cand1, Psma5, and finally to Ola1 and Rpl13 towards the far right, indicating their lower abundance in the sample set. The density distribution in protein expression levels among replicates and time points is shown in Figure S4. Correlation Heatmap of Protein Intensities is further elucidated in Figure S5, showcasing the complex interplay between various proteins in the disease state.

**Figure 4:**
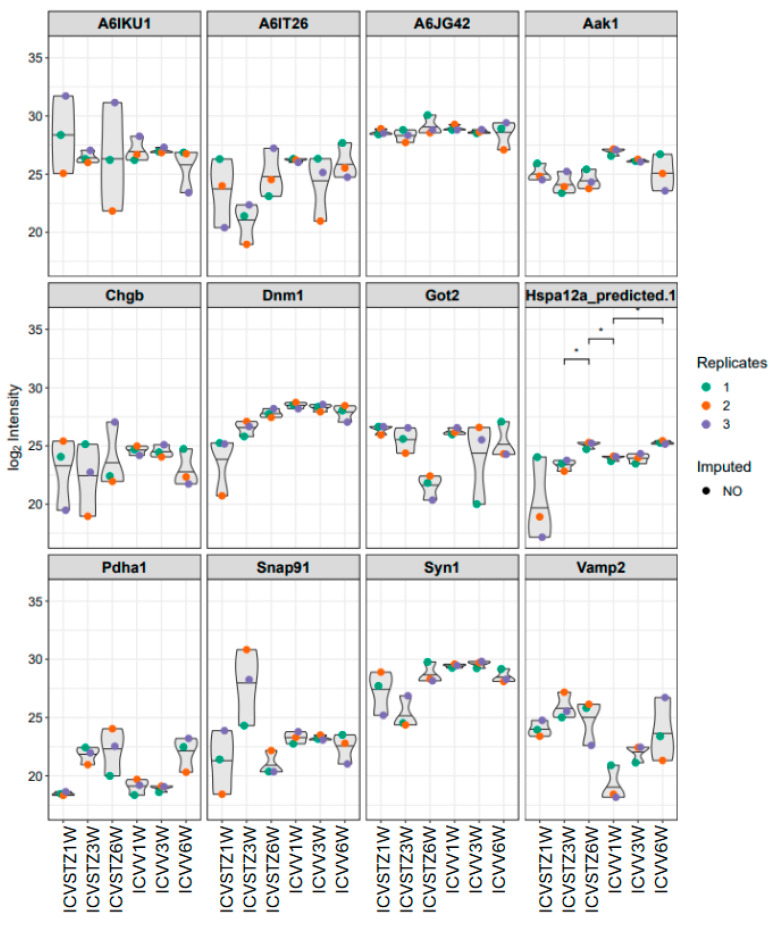
Violin plots representing the distribution of log2-transformed intensity values for selected proteins. Each plot corresponds to a specific protein, with individual points denoting replicate measurements—colored as red for replicate 1, orange for replicate 2, and blue for replicate 3. Gray shapes indicate the overall distribution of values, while colored points reflect observed data; the absence of a colored dot indicates imputed data points for missing values. Asterisks (*) in the figure indicate statistical significance between the conditions compared (*p* < 0.05). Statistical significance was determined using ANOVA followed by post-hoc analysis for multiple comparisons.

In the analysis of proteomic changes, a consistent expression pattern across control and ICV-STZ treated samples at W1, W3, and W6 is observed for proteins such as A6IKU1, A6IT26, and A6JG42, indicating their potential role in processes unaltered by ICV-STZ treatment. In contrast, Hspa12a (heat shock protein family A (Hsp70) member 12A) shows significant variability, suggesting a differential regulation upon treatment (adj. *p*-value < 0.05) (Figure 4). Pyruvate dehydrogenase E1 component subunit alpha (Pdha1) demonstrates increased expression levels over time, which may indicate progressive alterations in metabolic pathways because of the ICV-STZ treatment. In contrast, proteins involved in synaptic functioning, such as Synapsin-1 (Syn1) and Vesicle-associated membrane protein 2 (Vamp2), exhibit a moderate variability in expression, reflecting the subtleties of synaptic alterations post-treatment.

**Figure 5:**
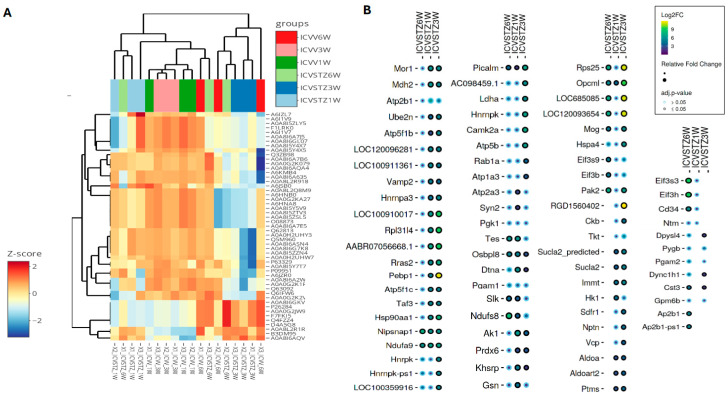
Hierarchical Clustering and Differential Expression Analysis in an STZ-Induced Alzheimer’s Model. Panel (**A**) presents a heatmap coupled with hierarchical clustering, showing proteins’ z-score normalized expression levels across six experimental groups, denoted by color coding at the top: ICV-STZ treated (1, 3, and 6 weeks) and their corresponding controls. Each row represents a different protein, while each column represents an experimental replicate—the clustering dendrograms on axes group proteins and replicates based on expression similarities. Panel (**B**) details a dot plot showing selected proteins’ log2 fold change (Log2FC) at each time point. Circle size indicates the relative fold change in abundance, and color coding signifies the adjusted *p*-value, with a smaller *p*-value represented by a darker shade. The statistical significance cutoff was set at an adjusted *p*-value of <0.05.

The heatmap visualizes the z-score normalized protein expression levels from ICV-STZ treated rat cortices samples at 1 W, 3 W, and 6 W post-treatment (Figure 5A). The z-score normalization emphasizes these shifts, with proteins that exhibit higher expression in the treated groups displaying a marked change towards red compared to the control group. Conversely, those with lower expression levels are represented in shades of blue, showing a decrease in abundance relative to the control. A consistent red across the 1 W, 3 W, and 6 W columns indicates sustained upregulation over the studied time course. The dot plot illustrates the differential abundance of proteins across the time points studied (Figure 5B). The size of the dots signifies the magnitude of change, with larger dots indicating a more pronounced fold change. The circle color reflects the adjusted *p*-value, with black denoting proteins with statistically significant changes (*p* < 0.05), whereas blue represents non-significant changes. Proteins such as Picalm, Nipsnap1, and Ndufa9 are indicated by larger black circles across multiple time points, suggesting these proteins undergo significant and consistent abundant changes in response to ICV-STZ treatment. Conversely, proteins with smaller blue circles, such as pgk1 and pgam1, show changes that do not reach statistical significance. Notably, some proteins exhibit a significant fold change at earlier time points (1 W or 3 W) but not at later stages (6 W) or vice versa. The important changes observed in proteins such as Pepbp1, Rps25, and RGD1560402 at W3, characterized by large yellow dots (>9Log2FC; adj. *p*-value < 0.05), suggest these proteins play crucial roles in the later stages of the model’s pathology (Figure 5B). The coefficient of variation (CV) distributions for each treatment and time point were measured, with median CVs annotated to provide insight into the precision of our measurements (Figure S6). Pairwise correlation analyses were performed, which depicted strong positive relationships between proteomic patterns at various time points, suggesting a consistent proteomic response to ICV-STZ treatment throughout the study (Figure S7). The comprehensive nature of our protein coverage is depicted through a stacked bar chart, illustrating the cumulative number of proteins identified across all samples (Figure S8).

### 2.4. Temporal Dynamics of Proteomic Alterations in ICV-STZ Treated Rats: GO and KEGG Pathway Analyses from Acute to Chronic Stages

**Figure 6:**
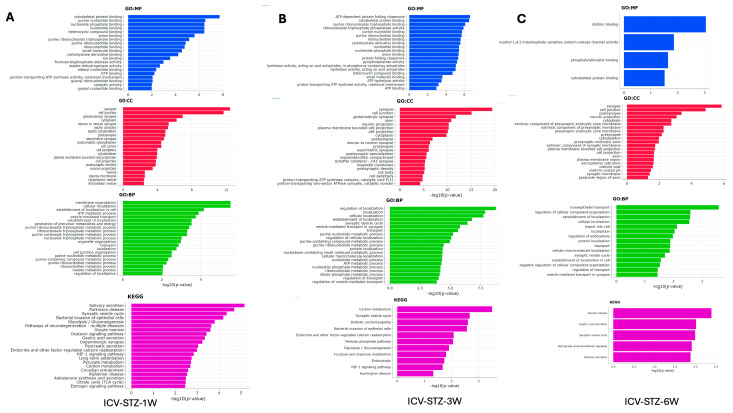
Gene Ontology and Pathway Enrichment Analysis in STZ-Induced Alzheimer’s Disease Model. Panel (**A**) represents the enriched Gene Ontology (GO) terms and Kyoto Encyclopedia of Genes and Genomes (KEGG) pathways in the 1-week post-treatment group. The x-axis displays the −log10 (*p*-value) for the most significant GO terms in the categories of molecular function (GO: MF, blue), cellular component (GO: CC, red), and biological process (GO: BP, green), along with KEGG pathway analysis (purple). Panel (**B**) shows similar enrichment analysis results for the 3-week post-treatment group. Panel (**C**) depicts the analysis for the 6-week post-treatment group, highlighting the most significant terms and pathways at this later stage. Bars extend rightward from the y-axis corresponding to their −log10 (*p*-value), indicating the significant level of enrichment for each term or pathway, with longer bars representing higher significance. The analyses elucidate the evolving biological context of the disease model over time, identifying critical molecular and cellular processes affected during the progression of Alzheimer’s-like pathology induced by STZ.

The gene ontology (GO) enrichment analysis and Kyoto Encyclopedia of Genes and Genomes (KEGG) pathway analysis elucidate the cellular functions and biological processes impacted in the AD model from 1 W to 6 W post-ICV-STZ treatment (Figure 6). Each bar in the charts represents a GO term or KEGG pathway, and the length corresponds to the −log10 (adj. *p*-value), which indicates the statistical significance of enrichment. At 1 W, molecular functions (GO: MF) related to cytoskeletal proteins and nucleotide binding are notably enriched, with −log10 (*p*-values) peaking around 8, indicating significant enrichment (Figure 6A). In cellular components (GO: CC), synapse-related terms such as neuron-to-neuron synapse and synaptic membrane show high enrichment (−log10 (*p*-value > 12). Biological processes (GO: BP) associated with vesicle-mediated transport and cell-cell junction organization are prominent, with −log10 (adj. *p*-values > 4). The KEGG pathway analysis highlights the glycolysis/gluconeogenesis pathway (−log10 adj. *p*-value > 3.5).

By 3 W, there is a noticeable shift, with (GO: MF) such as ATP binding and protein folding peaking around −log10 (*p*-values) of 6 (Figure 6B). Cellular components like the neuron projection and synaptic membrane remain highly represented, with *p*-values around the −log10 (*p*-value) of 15. Biological processes continue to show significant enrichment in metabolic processes and vesicle-mediated transport, with −log10 (*p*-values) around 7.5. KEGG pathways such as synaptic vesicle cycle and carbon metabolism show increased enrichment, with −log10 (*p*-values) around 3. At week 6 (Figure 6C), molecular function categories shift slightly, with cytoskeletal protein binding and phosphatidylinositol binding showing significant enrichment at a −log10 (*p*-value) of approximately 3. Cellular components such as the postsynaptic membrane and dendrite part continue to be enriched, with *p*-values peaking at a −log10 (*p*-value) of 6. For biological processes, terms related to vesicle-mediated transport in synapses show enrichment, albeit with a lower −log10 (*p*-value) of around 1, suggesting a decrease in significance. KEGG pathway analysis indicates that processes like synaptic vesicle cycle and retrograde endocannabinoid signaling are enriched, with a −log10 (*p*-value) close to 2.

### 2.5. Integrated Bioinformatics and Machine Learning Analysis Unveils Key Molecular Pathways in Disease Progression and Potential Therapeutic Targets

**Figure 7:**
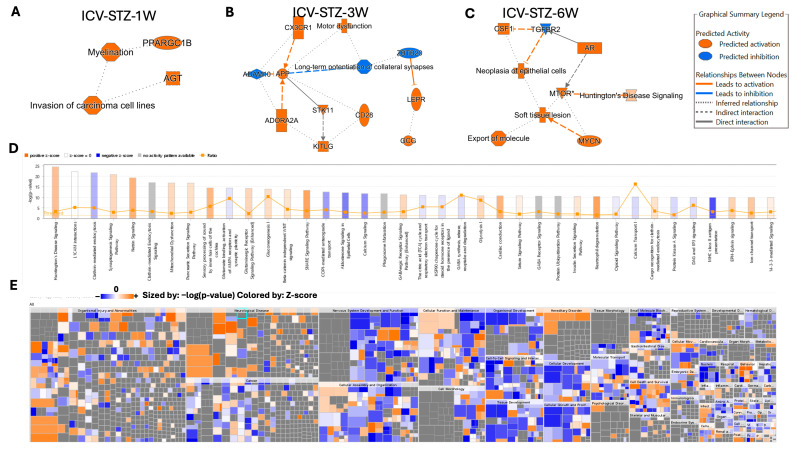
Ingenuity Pathway Analysis (IPA) of Molecular and Cellular Functions Altered in an STZ-Induced Alzheimer’s Disease Model Utilizing Machine Learning. Panels (**A**–**C**) illustrate the IPA-derived network analyses at 1, 3, and 6 weeks post-intracerebroventricular STZ administration, highlighting vital biological functions and diseases with the predicted activation (orange) or inhibition (blue) states based on the expression data. The graphical summary legend details the nature of the molecular relationships, including direct and indirect interactions and predicted activations and inhibitions, inferred through machine learning algorithms from IPA. Panel (**D**) shows a histogram of the z-scores for the top disease and function annotations across the time points, with orange representing a positive z-score, blue a negative z-score, and gray indicating no activity pattern available. Panel (**E**) displays a heat map of the disease and function annotations, with the intensity of the color representing the degree of association as determined by IPA’s machine-learning analysis. This multifaceted approach elucidates the complex biological landscape of the disease model, predicting the activation state of various pathways and functions implicated in the disease’s progression.

In our investigation into the molecular pathways of AD progression, we employed Ingenuity Pathway Analysis (IPA) software (v.24.0.1) to analyze and interpret the data generated from our proteomic experiments. Utilizing IPA’s algorithms, we aimed to uncover the intricate web of biological interactions and pathways affected in our model. By applying IPA’s network generation based on principles of biological relevance, interconnectivity, density optimization, and network size, we constructed biologically relevant networks from our list of differentially expressed proteomes. IPA’s causal analytics algorithms, including Upstream Regulator Analysis (URA), Mechanistic Networks (MN), Causal Network Analysis (CNA), and Downstream Effects Analysis (DEA), allowed us to predict upstream regulatory molecules, draw connections between regulators and genes within our dataset, and hypothesize on the broader implications of our findings on biological functions and diseases (Figure 7). The network depicted in ICV-STZ-1W reveals interactions between PPARGC1B and AGT, which are associated with myelination and the invasion of carcinoma cell lines (Figure 7A). PPARGC1B, a gene known to play a role in energy metabolism, is implicated in myelination, suggesting a potential link between metabolic processes and neuronal integrity. AGT is connected to the concept of carcinoma cell invasion, which might reflect an aberrant activation of processes typically associated with cell migration and tissue invasion.

ICV-STZ-3W treatment showed proteins such as CX3CR1 and APP, both of which are connected to the function of long-term potentiation of collateral synapses, indicating synaptic plasticity changes. CX3CR1 is also tied to motor dysfunction, while APP’s cleavage product generated by ADAM10 is implicated in AD. The involvement of STK11 and CD28 highlights the complexity of intracellular signaling pathways that may underline synaptic modifications and neuronal communication in the disease state (Figure 7B). ICV-STZ-6W treatment revealed that CSF1 and TGFBR2 are linked to neoplasia of epithelial cells, while the MTOR protein is associated with Huntington’s Disease signaling and soft tissue lesions. The direct interaction between MTOR and AR and the indirect association with MYCN suggest an involvement of growth and neurodegenerative disease signaling pathways, potentially reflecting the multifaceted nature of Alzheimer’s disease that goes beyond classical neurodegeneration (Figure 7C).

The histogram in Figure 7D reflects the −log10 (*p*-value) of the involvement of various pathways, with the threshold line demarcating the significance level. Pathways such as Huntington’s Disease Signaling and synaptogenesis signaling exhibit positive z-scores above the threshold, indicating a significant activation in our dataset (Figure 7D). This is consistent with the neuronal and synaptic dysfunctions commonly observed in AD. Conversely, pathways like Glutamate Receptor Signaling and Calcium Signaling, crucial for neuronal signaling and plasticity, show negative z-scores, suggesting a significant downregulation in these pathways. The interconnected pathways and regulatory networks are elaborated in Figure S9.

Applying IPA’s Knowledge Mining has allowed the identification of approximately 1500 diseases, phenotypes, and function pathways (Figure 7D). For example, the high positive z-score observed in the Glycolysis/Gluconeogenesis pathway aligns with altered energy metabolism associated with AD. The machine learning-driven summary construction has helped to infer relationships and visualize biological activities, such as the activation of microtubule dynamics, which are not directly observed but are crucial to understanding the disease’s mechanistic landscape. The heatmap in Figure 7E complements the previous analysis by providing a high-resolution view of the disease and biological function categories affected in our AD model. Sized by the −log10 (*p*-value) and colored by the z-score, the heatmap presents an intricate portrait of the biological landscape modulated by the disease state. Notable categories such as ‘Organismal Injury and Abnormalities’ and ‘Neurological Disease’ are prominently featured with both high significance and positive z-scores, emphasizing the pronounced impact of AD pathology on these functions. In contrast, areas like ‘Cancer’ show a mixture of positive and negative z-scores, suggesting a complex interplay of biological processes that the disease or treatment regimen could influence.

Further investigation within ‘Nervous System Development and Function’ reveals significant activation across multiple facets of this category, aligning with the expected neurodegenerative aspect of AD. ‘Cellular Function and Maintenance’ and ‘Cell-to-Cell Signaling and Interaction’ exhibit a range of activations and suppressions, reflecting the disruption of cellular homeostasis and communication pathways in the diseased state. Categories like ‘Cell Death and Survival’ and ‘Cellular Growth and Proliferation’ depict a nuanced pattern, with patches of significant activation (orange squares) interspersed with areas of no significant change (grey squares), indicating selective regulation of these processes at various stages or conditions of the disease model. Overall, the heatmap visualizes specific disease mechanisms and biological functions that are dysregulated in AD, offering a quantitative foundation for understanding the model’s complexity and guiding further experimental inquiry into these targeted areas (Figure 7E).

## 3. Discussion

This study presents a widespread temporal analysis of proteomic changes in an ICV-STZ-induced AD model in Wistar rats, elucidating the dynamic progression of protein expression in the cortex—a primary site of AD pathology [23]. Our longitudinal proteomic analysis offers a detailed temporal examination, uncovering the progression from early metabolic dysfunction to later-stage synaptic and cytoskeletal alterations. Using both data-independent acquisition (DIA) and data-dependent acquisition (DDA) mass spectrometry techniques has unveiled significant alterations in protein expression and perturbed pathways, which are critical to understanding the pathogenesis of AD. Through a meticulous experimental protocol, this study delineates the progressive stages of AD-like pathology at one, three-, and six weeks post ICV-STZ administration [24]. Noteworthy is the emphasis on a refined stereotaxic delivery of STZ, calibrated to induce the characteristic oxidative stress and cognitive impairments associated with AD without peripheral glucose imbalances [25].

The differential protein abundance observed across various stages of disease progression (Figure 2) suggests a multifaceted response to ICV-STZ treatment. The initial downregulation of proteins such as Aldoa and Pgk1 related to energy metabolism and synaptic structure during the first week post-administration underscores the metabolic hypothesis of AD etiology [26,27,28]. This decline is characterized by impaired glucose utilization and reduced energy metabolism, as described by Vijay et al. [29]. Furthermore, the upregulation of stress response proteins like Heat shock proteins (Hspa1b), observed at the three-week mark, aligns with studies [30,31,32,33] that discussed the activation of cellular stress responses during AD progression. The observed temporal downregulation of synaptic proteins, such as Synapsin-1 (Syn1) and Vesicle-associated membrane protein 2 (Vamp2), provides a proteomic narrative that complements the synaptic dysfunction widely reported in AD literature, akin to the synaptic loss [34,35,36,37].

The downregulation of Vimentin (Vim) across the six-week timeframe reflects the reactive gliosis found in AD, as reported in [38]. Vimentin and Peroxiredoxin-6, protein level changes associated with neuroinflammation, and neuronal stress response illustrate a shift towards chronic pathological responses (Figure 2F) [39,40,41]. The downregulated presence of these proteins by the sixth week corroborates their proposed role in neurodegenerative processes and reinforces the relevance of addressing oxidative stress in therapeutic strategies [38,42].

Moreover, the differential expression of the mitochondrial electron transport chain components, such as NADH-ubiquinone oxidoreductase 75 kDa subunit (Ndufs1), highlights mitochondrial dysfunction as a critical element of AD pathophysiology [43,44,45]. These findings echo the mitochondrial cascade hypothesis [46], which posits mitochondrial dysfunction as a primary event leading to AD. Future research should explore strategies to bolster mitochondrial function and oxidative stress resilience, offering a therapeutic avenue to mitigate AD progression. Our data also indicated alterations in proteins associated with neuroinflammation and glial cell activation, such as Glutamine Synthetase (Glul), predominantly expressed in astrocytes [47,48]. The role of glia in AD, especially astrocytes and microglia, has gained significant attention, with these cells implicated in both protective and degenerative processes within the AD brain [49,50,51]. The observed proteomic signatures suggest that glial cells undergo functional changes throughout AD, potentially shifting from a neuroprotective to a neurotoxic role. Investigating the triggers and consequences of glial activation in AD could reveal targets for modulating neuroinflammation and halting disease progression.

Ingenuity Pathway Analysis (IPA) further enriched our understanding by elucidating the interconnected pathways affected by the disease state. The machine learning components of IPA identified significant alterations in canonical pathways, such as Huntington’s Disease Signaling and Glycolysis/Gluconeogenesis, which parallel the impaired energy metabolism and synaptic deficiencies often seen in AD [52] (Figure 7D). Moreover, the inferred relationships generated by IPA’s machine learning algorithms enabled the identification of novel pathways that might not be immediately apparent through traditional analysis (Figure 7E). The involvement of PPARGC1B in myelination and AGT in the invasion of carcinoma cell lines suggests that pathways traditionally associated with other biological processes may also play a role in AD pathogenesis. This finding may reflect the complexity of AD, indicating potential shared mechanisms with other neurodegenerative diseases and even cancer biology [53,54] (Figure 7A).

The disease and biological function categories illustrated in the heatmap (Figure 7E) delineate the extensive impact of AD pathology. Notable areas such as ‘Organismal Injury and Abnormalities’ and ‘Neurological Disease’ exhibited high significance and positive z-scores, emphasizing the extensive influence of AD pathology on these functions. Our discussion integrates these findings into the current landscape of AD research, highlighting the potential of a proteomics-based approach in identifying novel pathways and targets. It also sets the stage for further validation studies and underscores the importance of integrating various omics data to form a holistic view of AD pathophysiology. Future research should explore the therapeutic potential of the identified targets and pathways, focusing on those proteins consistently altered across all examined time points. It is crucial to expand upon the machine learning analyses to predict the activation state of various pathways and functions implicated in disease progression, ultimately informing the development of intervention strategies [55].

While our study provides valuable insights into the molecular mechanisms of AD using an ICV-STZ-induced rodent model, several limitations must be acknowledged regarding the translational potential of these findings to human AD pathology. First and foremost, relying on a single animal model to mimic human AD pathology is a significant constraint, considering the complexity and heterogeneity of the disease in humans. The ICV-STZ model primarily simulates aspects of sporadic AD related to metabolic dysfunction, which may not encompass the entire spectrum of pathological events seen in human patients. Rodents have a less developed prefrontal cortex and different immune system responses, which may not fully replicate the chronic neuroinflammation in human AD [14,56]. Another limitation lies in the exclusive use of male Wistar rats. This gender-specific approach does not account for the potential differences in disease pathology and progression that could be present in females, which is particularly pertinent given that AD prevalence is higher in women [57,58]. The utilization of female subjects in subsequent studies is paramount, considering the gender disparities in AD prevalence. Such inclusion would enhance the generalizability of our findings and uncover potential sex-specific therapeutic targets. Finally, while our study spanned six weeks post-treatment, more is needed to observe the entire progression of the disease, especially for late-stage pathological events. A longer duration might provide additional insights into the chronic phase of the disease and further clarify the relevance of the observed protein changes.

These limitations stress the need for a multi-faceted approach, including different AD models, genders, broader omics analyses, and longer observation periods, to validate and extend our findings. Such comprehensive studies will help to translate these findings into clinical research and, eventually, therapeutic interventions for AD. To ensure the translational validity of our findings, we plan to perform cross-species validations using transgenic mouse models. This will bolster the clinical relevance of our identified protein targets and pathways. In parallel, integrating our proteomic data with genomics, transcriptomics, and metabolomics is expected to unravel complex molecular networks and regulatory mechanisms in AD (Figure 7). An in-depth examination of post-translational modifications (PTMs) is another critical aspect that can illuminate the functional consequences of protein alterations observed in our study, offering insights into regulating protein activity and interactions in AD. The advancement of machine learning algorithms will be integral to refining predictive disease progression models, enhancing our findings’ predictive power and clinical applicability. Such technological developments could pave the way for personalized medicine approaches in AD management.

In conclusion, our work provides a valuable resource for the AD research community, offering a rich dataset of protein expression changes. By delineating the trajectory of proteomic fluctuations, we pave the path for future investigations to explain the molecular underpinnings of AD further and spearhead the development of novel therapeutics aimed at the early and precise interception of the disease.

## 4. Materials and Methods

### 4.1. Reagents and Equipment

High-purity reagents essential for proteomic analysis were procured, including protease inhibitor tablets and BCA protein assay kits from Thermo Fisher Scientific Inc. (Pittsburgh, PA, USA). Acetonitrile and formic acid were sourced from Sigma-Aldrich (St. Louis, MO, USA). High-grade trypsin (Promega Corporation, Madison, WI, USA), Iodoacetamide (IAA), and dithiothreitol (DTT) (Sigma-Aldrich, St. Louis, MO, USA) were also obtained. Equipment critical for protein isolation and analysis, including a high-speed centrifuge, EASY-nLC™ 1200 system, and Q-Exactive HF LC-ESI-MS/MS system, was calibrated and validated for proteomic studies.

### 4.2. Animal Model and Tissue Preparation

Three-month-old adult male Wistar rats were utilized to establish an intracerebroventricular streptozotocin (ICV-STZ) induced model of Alzheimer’s disease (AD), focusing on the brain’s cortical region for proteomic analysis. The Animal Resources Center at the Faculty of Medicine, Kuwait University, provided the animals, and they were housed under controlled conditions conducive to their well-being. Following the guidelines of the Health Sciences Center Animal Research Ethics Committee at Kuwait University, which align with the NIH Guidelines for the Care and Use of Laboratory Animals, the rats underwent surgery for administration. The procedure was designed to minimize distress and ensure the delivery of STZ precisely into the lateral ventricles, fostering the development of an Alzheimer’s-like pathology in the prefrontal cortex (PFC).

### 4.3. Experimental Design

The AD model was established using intracerebroventricular streptozotocin (ICV-STZ) to induce oxidative stress and cognitive impairments in the brain, and the temporal progression of these alterations was emphasized. The study was conducted anonymously to minimize bias, with animals randomly assigned to each experimental group.

Sixty adults male Wistar rats were divided into six groups, with ten rats per group, representing different time points post-administration: one week (ICVV-1W and ICV-STZ-1W), three weeks (ICVV-3W and ICV-STZ-3W), and six weeks (ICVV-6W and ICV-STZ-6W). Control groups received 5 µL of sterile citrate buffer (0.1 M, pH 4.6) into each lateral ventricle, mirroring the ICV-STZ groups in which rats were injected with streptozotocin (3 mg/kg body weight) in sterile 0.1 M citrate buffer, adjusted to pH 4.0, and made in artificial cerebrospinal fluid (CSF). This specific STZ dose is based on previous studies [4,59,60].

Following injections, rats were observed for 1, 3, or 6 weeks. ICV-STZ-treated rats did not exhibit elevated blood glucose levels, reinforcing the localized effect of the treatment. At the end of each observation period, rats were euthanized, and their brains were rapidly harvested. Given its significance in AD research and for proteomic analyses, the focus was on isolating the prefrontal cortex. Cortical tissues were immediately snap-frozen in liquid nitrogen and preserved at –80 °C until processed for detailed proteomic studies.

#### Surgical Procedure for ICV-Vehicle/STZ Injection

The surgical procedure for intracerebroventricular (ICV) administration of vehicle or streptozotocin (STZ) was meticulously carried out under aseptic conditions to ensure minimal animal distress, following protocols established in prior studies [60]. Before the operation, all surgical tools and the stereotaxic apparatus were sterilized using autoclaving and 70% ethanol to maintain a sterile environment.

For anesthesia, rats received an intramuscular injection of a ketamine hydrochloride/xylazine hydrochloride solution (ketamine: 60 mg/kg, xylazine: 5 mg/kg). Once anesthetized, each rat was secured in a stereotaxic frame. A small incision was made on the scalp to expose the skull, and two marks were placed for the creation of burr holes: 0.8 mm posterior to the bregma and 1.5 mm lateral to the midline on both sides. Care was taken to drill these holes up to the dura mater without causing damage to the underlying brain tissue.

A 10 µL Hamilton syringe equipped with a 26 G needle, filled with either sterile citrate buffer (0.1 M, pH 4.7) for the control group or STZ solution prepared in the same buffer for the ICV-STZ group, was inserted 4.0 mm below the dura mater to target the lateral ventricles accurately. For the control group, 5 µL of sterile citrate buffer was administered into each ventricle over 20 min to ensure optimal diffusion without local toxicity. Similarly, the ICV-STZ group received 5–6 µL of STZ solution (1.5 mg/kg/ventricle) in sterile citrate buffer over 20 min. To prevent the backflow of the injected solution, the syringe was left in place for an additional five minutes before being carefully withdrawn.

Post-injection, the incision was sutured, and a betadine solution was applied to the site for its antiseptic properties. Each rat was placed in a warmed recovery area until it regained consciousness. Post-operative care included the daily application of betadine for three days and providing wet food within the cage. Rats were housed individually for the remainder of the study to monitor recovery and ensure well-being.

### 4.4. Post-Treatment Brain Tissue Processing for Proteomic Analysis

Following the treatment periods of 1, 3, and 6 weeks, rats from all experimental groups were euthanized for brain tissue collection. To eliminate bias, tissue samples were anonymized by a third party before processing. The cortex was rapidly dissected, immediately snap-frozen in liquid nitrogen, and stored at –80 °C. This preservation step is crucial for maintaining the integrity of the proteome for subsequent analysis. The stored tissues were then prepared for proteomic workflows involving isolating protein samples for mass spectrometry-based analyses to identify and quantify differential protein expressions related to Alzheimer’s disease pathology. Cortical brain tissues were homogenized using a Wheaton tissue homogenizer in a chilled homogenization buffer of 8 M urea and 50 mM ammonium bicarbonate to preserve protein integrity. The homogenate was then subjected to brief sonication to reduce sample viscosity, followed by centrifugation at 16,000× *g* for 10 min to remove debris.

Protein concentrations in the supernatant were determined using a BCA assay. A quantity of 20 µg of total protein from each sample was then reduced by incubation with 10 mM dithiothreitol (DTT) at 60 °C for 30 min. The samples were allowed to cool to room temperature, followed by alkylation with 25 mM iodoacetamide (IAA) for 25 min in the dark to prevent interference with the alkylation reaction. For reduction and alkylation, dithiothreitol (DTT) was added to a final concentration of 5 mM, and the mixture was incubated at 60 °C for 30 min, followed by alkylation with 15 mM iodoacetamide (IAA) in the dark at room temperature for 20 min. The sample was then diluted with 50 mM ammonium bicarbonate to reduce the urea concentration to less than 2 M before enzymatic digestion. Trypsin was added at a 1:50 trypsin-to-protein ratio and incubated at 37 °C for 16 h for protein digestion. This step was followed by centrifugation at 18,000× *g* for 10 min to precipitate any insoluble material. The reaction was quenched with formic acid to a final pH of <3. Peptides were desalted using a C_18_ solid-phase extraction column and dried using a vacuum centrifuge. Before LC-MS/MS analysis, the peptides were reconstituted in 0.1% formic acid. The samples were analyzed by nanoLC-MS/MS using a Nano LC (nLC1200) system coupled to a high-resolution Orbitrap mass spectrometer.

### 4.5. DIA Acquisition and Data Processing

Peptides derived from cortical brain tissue homogenates were subjected to high-resolution analysis using the Bruker timsTOF Pro system coupled with a nanoElute High-Performance Liquid Chromatography (HPLC) system. Chromatographic separation of the peptides was carried out on a specialized C_18_ column featuring a particle size of 1.6 µm and dimensions of 75 µm by 25 cm. The separation was achieved through a carefully optimized gradient of water and acetonitrile, each containing 0.1% formic acid, over 90 min. This setup was chosen for its ability to provide a high peptide separation efficiency and resolution, which is crucial for the subsequent mass spectrometry analysis.

In the data-independent acquisition (DIA) phase on the Bruker timsTOF Pro, the mass spectrometer was operated leveraging the advanced capabilities of parallel accumulation serial fragmentation (PASEF) technology. This enabled the efficient and rapid acquisition of MS/MS data by employing expansive isolation windows to sequentially capture and fragment all precursor ions across the targeted *m*/*z* range of 400 to 1200. The instrument settings, including ramp time and collision energy, were optimized based on precursor ion characteristics. The ramp time was optimized to 100 milliseconds (ms) for each DIA window. Collision energy settings were intricately tailored based on precursor ions’ mass and charge state, applying a dynamic range of 20 to 40 electron volts (eV). For lower mass ions (*m*/*z* < 600), lower collision energy near 20 eV was utilized to prevent excessive fragmentation, maintaining identifiable peptide fragments. For higher mass ions (*m*/*z* > 1000), the collision energy was increased up to 40 eV to ensure adequate fragmentation. The overall cycle time was carefully calibrated to scan each DIA window multiple times throughout the LC run.

The raw DIA data was processed and analyzed using Bruker’s Compass Data Analysis software (v. 4.4), focusing on feature detection and extraction to ensure high-quality data were forwarded for peptide identification. A spectral library, generated from data-dependent acquisition (DDA) runs or curated from relevant databases, is a reference for identifying peptides by matching the DIA spectra against library entries. The quantification of peptides involved using specialized software, Spectronaut (v. 18), which relied on extracted ion chromatograms (XICs) for every identified peptide.

### 4.6. Label-Free Data-Dependent Acquisition LC-ESI-MS/MS

Samples underwent analysis utilizing the Q-Exactive HF LC-ESI-MS/MS system from Thermo Fisher Scientific, paired with an EASY-nLC™ 1200 nano-LC System through an EASY-Spray Ion Source (Thermo Fisher Scientific). The mobile phase A comprised 0.1% formic acid in water, while mobile phase B consisted of 0.1% formic acid in 80% acetonitrile and water. For the LC separation, a trap-elute configuration was utilized, integrating both a trapping column (Acclaim PepMap100, 75 μm × 2 cm) and an analytical column (ES801A PepMap RSLC C18, 2 μm, 100 A, 50 μm × 15 cm), both from Thermo Fisher Scientific. The process began with loading the trapping column with 5 μg of digested peptide, using mobile phase A at a delivery rate of 5 μL/min for 3 min. This step was designed to trap and purify the peptides. The samples were transferred to the analytical column at a 300 nL/min flow rate. The eluent was ionized using an Easy Spray nano ESI source operating in positive ion mode with a nanoflow column maintained at 40 and a run duration of 170 min. The ionization voltage was 2.3 kV, and the capillary temperature was 230 °C. Data-dependent acquisition (DDA) mode was used to operate the Q Exactive HF, and it was automatically switched between full scan MS and MS/MS acquisition. MS1 full scans were acquired for 350–2000 *m*/*z* at 120,000 resolution, 60 ms maximum IT, 3 × 10⁶AGC target. The top 10 multiply charged ions were chosen for MS/MS analysis, where they were fragmented using higher-energy collisional dissociation (HCD). The process was set with a normalized collision energy of 27 eV. This was conducted at a resolution of 30,000, with a maximum injection time (IT) of 20 ms, an automatic gain control (AGC) target of 1e5, and an isolation window of 2.0 *m*/*z*. 

### 4.7. Workflow for Label-Free Quantitative Proteomics Analysis

The protein quantification in this workflow was conducted using Thermo Scientific’s Proteome Discoverer software (version 3.1.0.638). Raw spectral data was obtained and preprocessed to ensure quality before analysis. The preprocessing involved using the “Spectrum Files RC” node for data retrieval, followed by the “Minora Feature Detector” for feature identification within the raw spectra. Selected features from the spectral data were processed through the Spectrum Selector node to filter and isolate relevant spectra. This was followed by the Precursor Detector node, which identified precursor ions in preparation for peptide identification. The filtered spectral data was subjected to peptide-spectral matching via three search algorithms: CHIMERYS, Sequest HT, and Comet. CHIMERYS was utilized as the primary search engine. A non-redundant protein sequence database (uniprotkb_taxonomy_id_10116.fasta) was employed, with trypsin as the specified enzyme for up to two missed cleavages. The peptide length was set between 7 and 30 amino acids, with a peptide charge range from 2 to 4. The precursor mass tolerance was set at 20 ppm, and the fragment mass tolerance at 0.02 Da.

Static modifications included oxidation on methionine residues and carbamidomethylation on cysteine residues, with up to three modifications allowed per peptide. Specific static modifications, including carbamidomethyl (C) at peptide N-termini, were also designated. Post-search validation was performed by the Target Decoy PSM Validator node, ensuring peptide-spectral matches met stringent criteria based on score thresholds defined by the search nodes and the target/decoy strategy. The False Discovery Rate (FDR) was set at strict (0.01) thresholds to validate identifications and ensure high-confidence results. The results from CHIMERYS, Sequest HT, and Comet searches were integrated to provide a comprehensive proteomic profile.

The quantification was based on a combination of unique and razor peptides, accounting for protein groups during peptide assignment. The software was configured to accept shared quantitative results and to exclude any with missing data. Quantitation was intensity-based with no minimum requirement for replicate features, ensuring comprehensive data inclusion. Normalization was performed on the total peptide amount, and scaling was done on the average of all detected peptides. All peptides were included for protein quantification and roll-up. The top *N* average method was applied, selecting the three most abundant peptides for protein abundance calculation. Ratio calculations were pairwise, accommodating a substantial fold change up to 1000. Imputation of missing values was handled by replicate-based resampling, and statistical significance was assessed via *t*-test. Results were categorized into distinct fold change thresholds ranging from 2-fold to 10-fold to facilitate detailed analysis of protein expression levels across the samples.

Stringent filtration criteria were applied to ensure high-confidence protein identification and quantification. Only proteins with 15 or more Peptide-Spectrum Matches (PSMs) and at least four unique peptides were considered to enhance identification accuracy—an adjusted *p*-value of 0.05 or less determined significance in protein abundance changes. The reliability of the peptide matches was further verified by a minimum Sequest HT score of 10. Consistency across biological replicates was ensured by including only proteins with detectable abundance in all samples. Differential protein expression was assessed with log2-transformed abundance ratios, accepting only those proteins exhibiting a change greater than or equal to a 2-fold increase or decrease. These criteria sharpened the dataset, focusing on the most biologically relevant and statistically robust proteins.

### 4.8. Bioinformatics Analysis: GO Enrichment and Network Analysis

Data visualization and analysis were facilitated using Amica [61] and LFQ-Analyst [62], respectively, to explore label-free quantitative proteomics data. Bioinformatics analyses such as GO enrichment and network analysis were performed using Ingenuity Pathway Analysis [63] (IPA QIAGEN, version 24.0.1), which employs machine learning to analyze molecular interactions and predictive markers within vast datasets. IPA facilitated in-depth canonical pathway analyses, disease and function assessments, regulator effects, identification of upstream regulators, and the construction of molecular networks. IPA’s network generation algorithm breaks down complex interaction maps into distinct networks, assigning each score based on hypergeometric distribution and the significance level derived from Fisher’s exact test. A −log (*p*-value) threshold greater than two was established for significance in canonical pathways and disease/function analysis. Activation states were determined with a Z-score, where values above 2 indicated significant activation and values below −2 signified significant inhibition. For regulator effects and molecular networks, consistency scores were employed, with higher scores reflecting more accurate predictions of regulatory impacts. Upstream regulators required a *p*-value of overlap below 0.05 to be considered significant. The algorithms for Z-score and *p*-value calculations adhere to previously established methods in the field.

## 5. Conclusions

This study investigates proteome changes over time in a Wistar rat AD model caused by ICV-STZ. It gives insights into the dynamic evolution of protein expression in the PFC of the brain. The results of our study reveal notable changes in the levels of proteins and disrupted pathways crucial for understanding the development of AD. The evolution of AD is characterized by early metabolic failure, activation of the stress response, and later-stage abnormalities in synaptic and cytoskeletal structures, highlighting the complex nature of the disease. Essential factors such as malfunctioning mitochondria, the route of synaptic vesicles, and signaling related to neuroinflammation were shown to be crucial components of the pathogenesis of AD. Although our work provides valuable insights, its scope is restricted due to using just one animal model and the exclusive inclusion of male rats. This may not completely encompass the intricacy and diversity seen in human AD. To improve the translational potential of our results, future research should include other AD models, involve both genders, use more comprehensive omics studies, and extend the observation durations. By combining proteomic data with genomes, transcriptomics, and metabolomics and doing cross-species validations, we may better understand the intricate molecular networks involved in AD. This holistic strategy will facilitate the creation of new biomarkers and therapeutic targets, eventually leading to the implementation of personalized medicine in treating AD.

## Figures and Tables

**Table 1:** Temporal Protein Expression Profiles in ICV-STZ Treated Alzheimer’s Disease Rodent Model. This table presents a comprehensive overview of the protein expression profiles in a rodent model of Alzheimer’s disease at different time points following intracerebroventricular streptozotocin (ICV-STZ) treatment compared to control (ICV-Vehicle). The columns display the gene symbol, the corresponding protein name, percentage coverage, number of peptide spectrum matches (PSMs), unique peptides identified, overall protein score, and the log2 fold changes in protein abundance between the time points of 3 weeks and one week (3 W/1 W), six weeks and one week (6 W/1 W), six weeks and three weeks (6 W/3 W), and each time point compared to its respective control (1 W/C, 3 W/C, 6 W/C). The fold change color coding reflects the direction and magnitude of expression changes: blue indicates downregulation, red indicates upregulation, and white represents no significant change. The extent of color saturation corresponds to the degree of fold change. Proteins are ranked by the magnitude of their expression changes and the statistical significance of their altered abundance at each post-treatment time point, facilitating the identification of potential biomarkers and insights into the molecular dynamics of disease progression.

Gene Symbol	Protein Name	Coverage [%]	# PSMs	# Unique Peptides	Score	3 W/1 W	6 W/1 W	6 W/3 W	1 W/C	3 W/C	6 W/C
Gprin1	G protein-regulated inducer of neurite outgrowth 1	26	123	24	197.43	−0.04	3.91	−0.8	−1.85	1.76	0.04
Pgk1	Phosphoglycerate kinase 1	25	48	11	86.72	0.3	−3.77	−4.04	1.72	2.7	−2.77
Atp6v1b2	V-type proton ATPase subunit B, brain isoform	18	70	10	111.94	0.99	1	4.17	0.43	2.25	1.74
Mbp	Myelin basic protein	25	88	10	158.79	1.79	5.15	−1.84	−0.84	3.9	2.29
Canx	Calnexin	9	52	7	83.15	−1.73	5.52	−0.66	−0.96	−1.03	−0.23
Glul	Glutamine synthetase	18	81	7	148.53	−2.77	0.36	5.3	−0.72	−1.92	1.45
Atp5f1c	ATP synthase subunit gamma	21	54	6	87.43	3.03	−3.3	−3.17	4.8	3.26	−0.98
Dlst	Dihydrolipoyllysine-residue succinyltransferase component of 2-oxoglutarate dehydrogenase complex, mitochondrial	13	21	6	34.1	2.43	4.19	0.39	0.12	−1.96	−1.4
Lsamp	Limbic system-associated membrane protein	13	54	5	82.43	−2.65	4.03	3.6	−0.16	−5.48	−3.73
Atp6v1g2	V-type proton ATPase subunit G	42	78	5	177.36	0.57	−4.49	−3.96	2.56	1.25	−4.48
Nucks1	Nuclear casein kinase and cyclin-dependent kinase substrate 1	20	19	5	32.98	4.05	1.44	−4.59	1.7	−0.29	−4.02
Prpsap2	Phosphoribosyl pyrophosphate synthase-associated protein 2	10	21	5	35.64	−2.24	−3.21	−2.57	0.56	−1.48	5.58
Got1	Aspartate aminotransferase, cytoplasmic	11	54	5	91.37	1.32	−2.53	0.52	2.57	3.09	−1.7
Ndufs1	NADH-ubiquinone oxidoreductase 75 kDa subunit, mitochondrial	7	21	4	36.45	−4.74	−1.05	3.59	0.39	0.35	0.54
Aldh1l1	10-formyltetrahydrofolate dehydrogenase	3	37	4	44.98	0.78	−4.06	−3.19	1.22	2.81	−1.97
Cap2	Adenylyl cyclase-associated protein 2	7	15	4	34.76	0.4	2.14	−0.56	1.84	0.58	2.36
Cadm2	Cell adhesion molecule 2	11	26	4	39.97	2.95	−0.98	−3.11	−2	−0.22	−1.85
	Myosin-6	2	24	4	39.3	1.01	−3.92	−4.13	2.26	1.63	−5.41
Crmp1	Collapsin response mediator protein 1	12	118	4	212.19	−0.58	−1.13	4.09	−0.82	−0.44	−0.27
Cdv3	CDV3 homolog	23	82	4	105.62	−0.97	0.68	1.54	−0.19	−1.47	2
Grb2	Growth factor receptor-bound protein 2	14	25	4	40.85	1.84	−2.49	−2.01	1.74	2.04	−1.83
Ensa	Alpha-endosulfine	24	20	4	33.57	−2.82	−0.68	0.26	6.64	6.64	1.72
Wfs1	WFS1	4	26	4	37.31	1.3	6.64	5.74	−5.63	−4.74	1.75

## Data Availability

RAW mass spectrometric data are provided upon reasonable request.

## Supplementary Materials

The following supporting information can be downloaded at: https://www.mdpi.com/article/10.3390/ijms25126469/s1.
