# Supplementary material for: Deciphering Early and Progressive Molecular Signatures in Alzheimer’s Disease through Integrated Longitudinal Proteomic and Pathway Analysis in a Rodent Model
Source: Int J Mol Sci. 2024 Jun 12;25(12):6469. doi: 10.3390/ijms25126469 (PMC11203991; doi:10.3390/ijms25126469)
Supplement: Supplementary file 1 [file ijms-25-06469-s001.zip › ijms-2995544-supplementary-proofreaded.pdf]

Supplementary Notes

Deciphering Early and Progressive Molecular Signatures in Alzheimer's Disease through Integrated Longitudinal Proteomic and Pathway Analysis in a Rodent Model

Hamad Yadikar [1,2,*], Mubeen A. Ansari [3], Mohamed Abu-Farha [4], Shibu Joseph [5], Betty T. Thomas [2] and Fahd Al-Mulla [4]

1 Department of Biological Sciences, Faculty of Science, Kuwait University, Sabah AlSalem University City, Kuwait City 13060, Kuwait
2 OMICS Research Unit, Research Core Facility, Faculty of Medicine, Kuwait University, Kuwait City, 13110, Kuwait; bety.tomas@ku.edu.kw
3 Department of Pharmacology and Toxicology, Faculty of Medicine, Kuwait University, Kuwait City 13110, Kuwait; mubeen.ansari@ku.edu.kw
4 Department of Translational Research, Dasman Diabetes Institute, Kuwait City 15462, Kuwait; mohamed.abufarha@dasmaninstitute.org (M.A.-F.); fahd.almulla@dasmaninstitute.org (F.A.-M.)
5 Department of Special Service Facility, Dasman Diabetes Institute, Kuwait City 15462, Kuwait;
shibu.joseph@dasmaninstitute.org
* Correspondence: hamad.yadikar@ku.edu.kw

Keywords/Search Terms: Intracerebroventricular Streptozotocin, Alzheimer's Disease Proteomics, Temporal Expression Profiling, Neurodegenerative Biomarkers, Pathway Dysregulation.

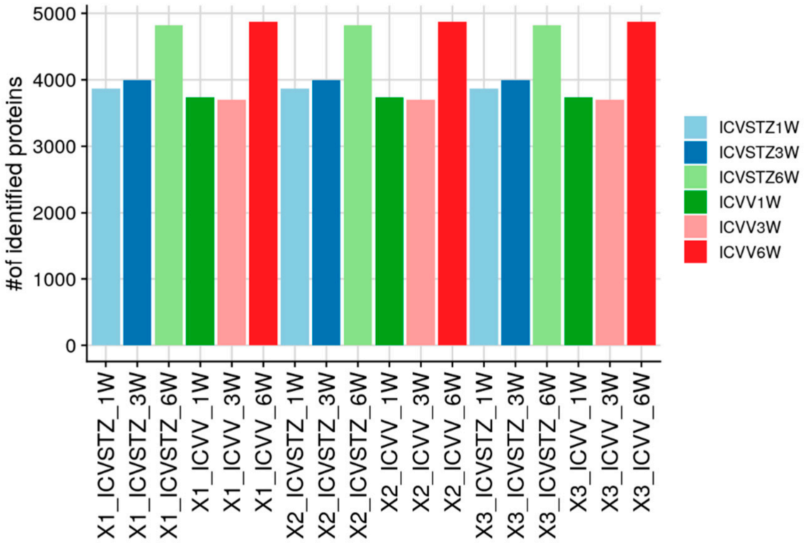

Figure S1: LFQ intensities before filtering and imputation determine the number of identified proteins. This bar graph shows the number of proteins identified at one week (1W), three weeks (3W), and six weeks (6W) post-administration in the intracerebroventricular streptozotocin (ICV-STZ)-treated (blue, green, and red) and vehicle-treated (ICVV) groups. Each bar shows a group's protein count at each time point, allowing temporal comparisons between the disease model and controls and throughout disease progression stages within each experimental condition. The data show that illness development and therapeutic effects modify the proteome landscape throughout time.

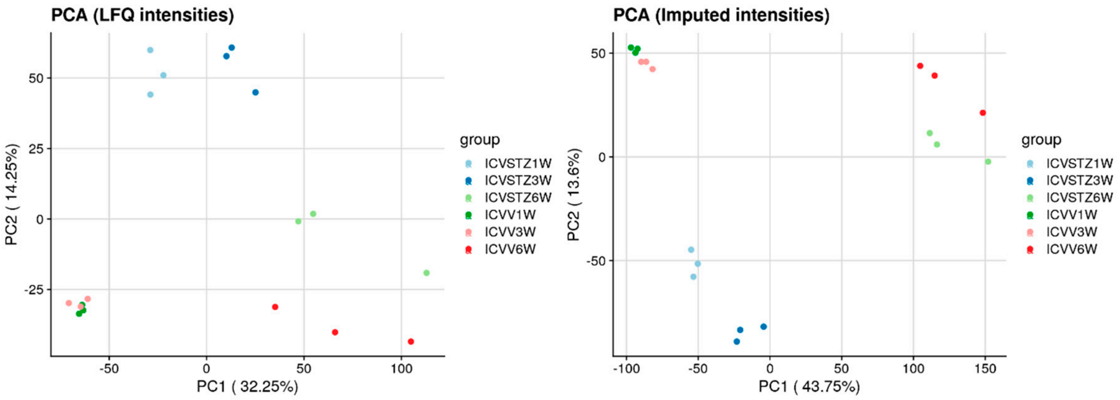

Figure S2: Principal Component Analysis (PCA) of Proteomic Data from ICV-STZ Alzheimer's Disease Model. The left panel shows the PCA of label-free quantification (LFQ) intensities and the right panel displays the PCA of imputed intensities. Each dot represents an individual sample, with colors indicating the experimental group: ICVSTZ1W (light blue), ICVSTZ3W (green), ICVSTZ6W (red) for the streptozotocin-treated groups, and ICVV1W (dark blue), ICVV3W (light green), ICVV6W (dark red) for the vehicle-treated controls. The percentage of variance explained by each principal component (PC) is denoted on the axes (PC1 and PC2).

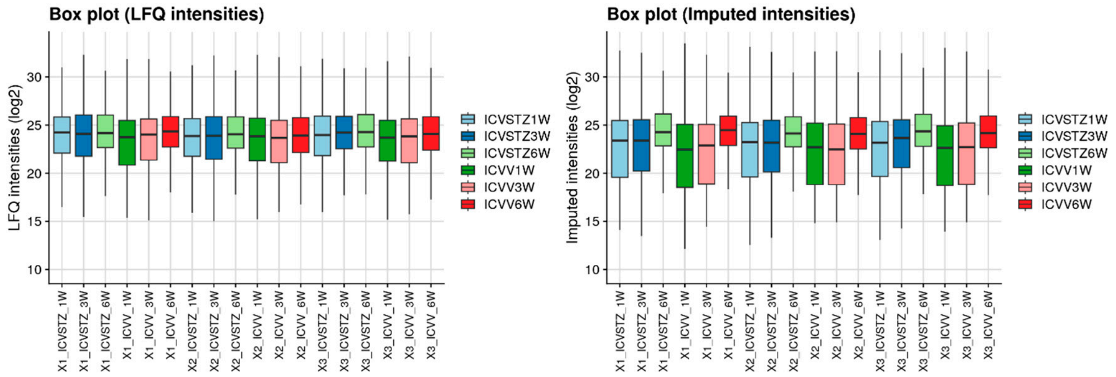

Figure S3: Box Plots of Proteomic Intensities in ICV-STZ Treated Alzheimer's Disease Rodent Model. The left panel illustrates box plots of the label-free quantification (LFQ) intensities, and the right panel presents the box plots of imputed intensities. Boxes represent the interquartile range (IQR) of the log2-transformed protein intensities for each experimental group, with the median value indicated by the line within each box. The colors correspond to the treatment and time points: ICVSTZ1W (light blue), ICVSTZ3W (green), ICVSTZ6W (red) for the streptozotocin groups, and ICVV1W (dark blue), ICVV3W (light green), ICVV6W (dark red) for the vehicle groups. Whiskers extend to 1.5 times the IQR from the box edges, highlighting the range of data and potential outliers. These plots provide a comparative view of the distribution and central tendencies of the proteomic data, reflecting the changes in protein expression levels across different time points and treatments within the study.

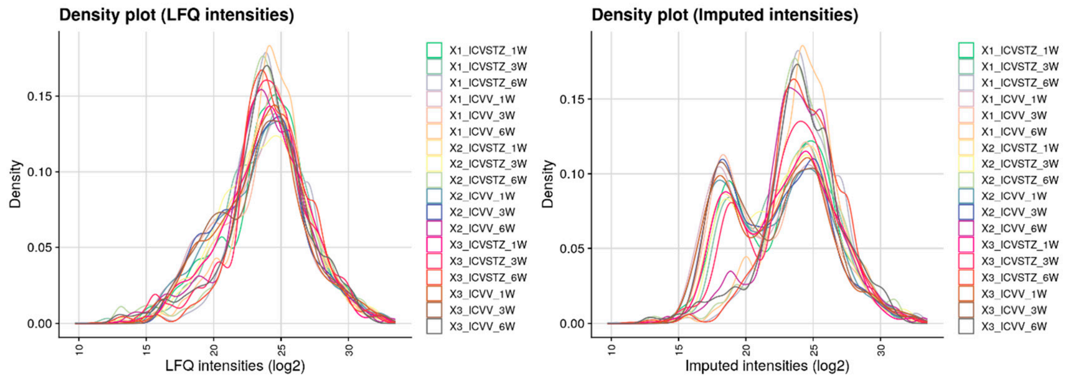

Figure S4: Density Distribution of Proteomic Data in ICV-STZ Treated Alzheimer's Disease Rodent Model. The figure displays two panels of density plots representing the distribution of protein expression levels. The left panel shows the density of label-free quantification (LFQ) intensities, while the right panel depicts the density of imputed intensities. The density provides a visualization of the distribution of log2-transformed protein intensities. The densities were calculated using kernel density estimation (KDE), a non-parametric method that approximates the probability density function of the data. KDE applies a smoothing kernel function, typically a Gaussian curve, with a set bandwidth to each data point and sums these contributions to produce a continuous density estimate over the range of the data. Each curve represents the kernel density estimation of the log2-transformed intensities for a specific group at a given time, allowing for the visualization of the data's distribution shape and spread. The line colors differentiate each group and time point combination.

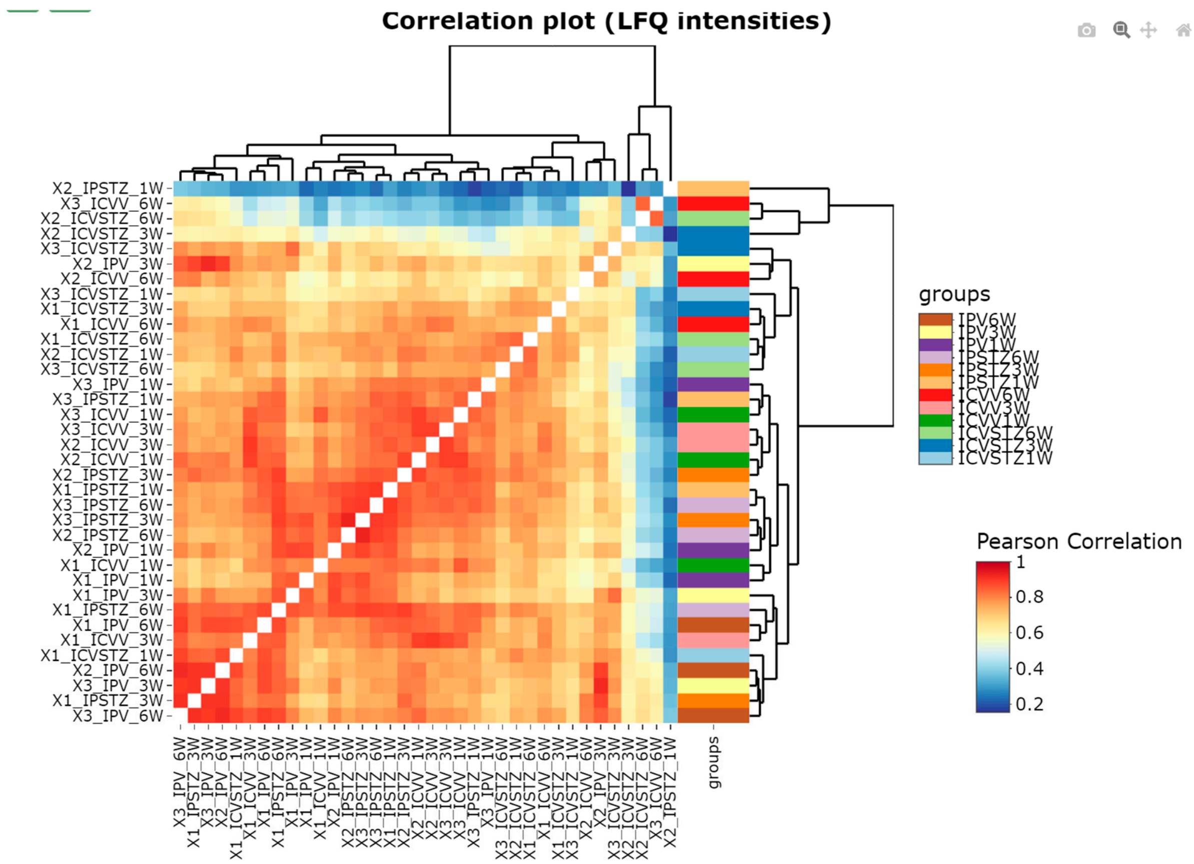

Figure S5: Correlation Heatmap of Protein Intensities. This heatmap visualizes the Pearson correlation coefficients calculated for the log2-transformed label-free quantification (LFQ) intensities of proteins across various treatment groups and time points in the ICV-STZ Alzheimer's disease rodent model. Each cell in the heatmap represents the correlation between pairs of samples, with the color intensity indicating the correlation strength—from blue (low correlation, Pearson r near 0) to red (high correlation, Pearson r near 1). Hierarchical clustering on both axes groups the samples with similar expression profiles together, aiding in identifying patterns and relationships within the data.

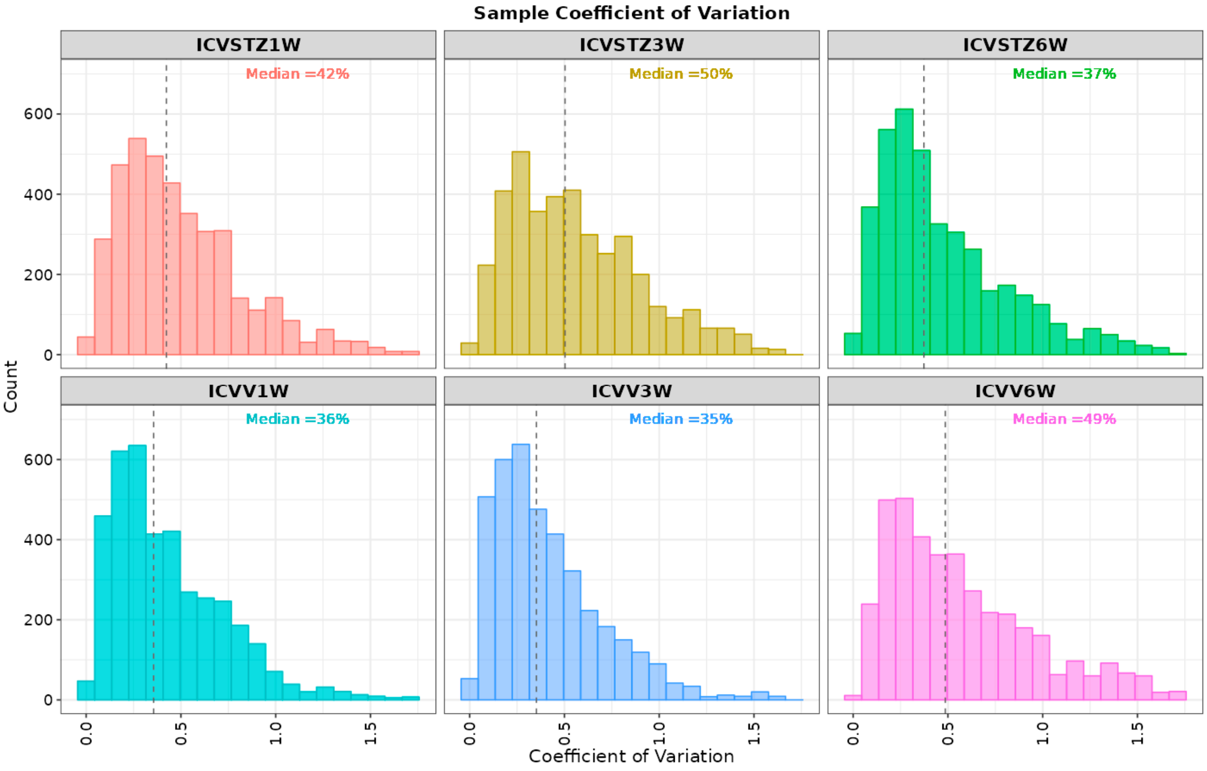

Figure S6: Distribution of Coefficients of Variation for Proteomic Quantification. Histograms representing the distribution of the coefficient of variation (CV) for protein quantifications from ICV-STZ treated (ICVSTZ) and vehicle-treated (ICVV) groups at 1, 3, and 6 weeks (W). Each panel displays the frequency distribution of CVs for the indicated treatment and time point, with the median CV annotated. These distributions provide insight into the protein expression variability within each sample set, highlighting the experimental reproducibility and data quality across different conditions.

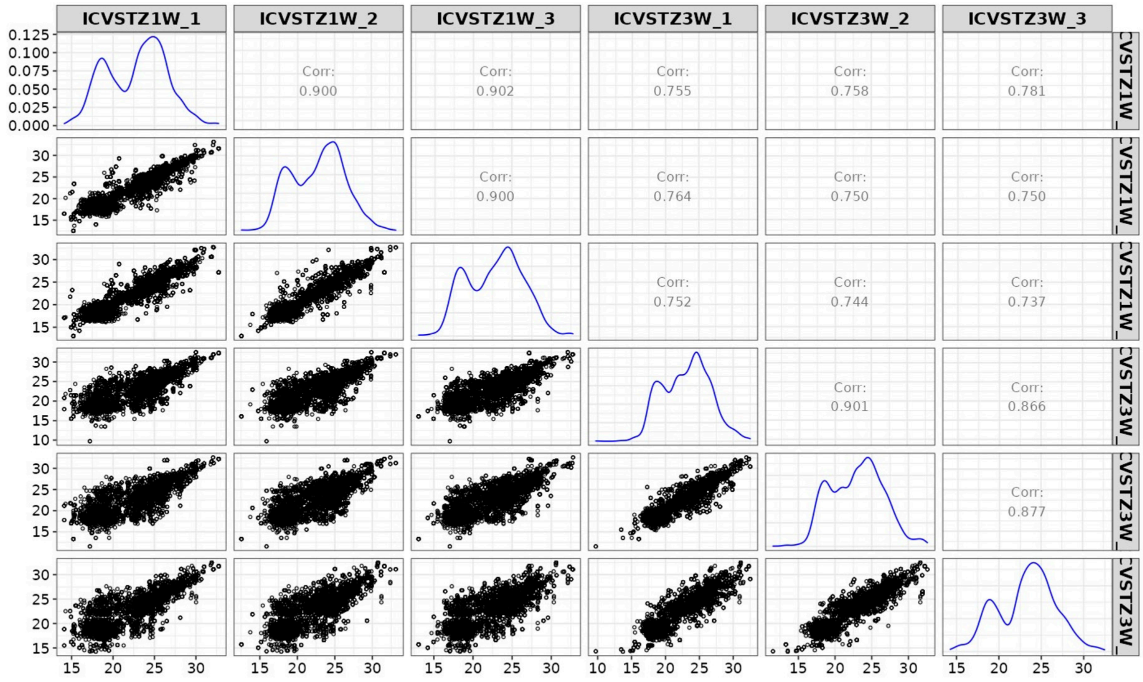

Figure S7: Pairwise Correlation of Proteomic Data Across Different Time Points in ICV-STZ Treated Groups. This figure represents a pairwise correlation matrix for proteomic data obtained from ICV-STZ treated rat models across various time points (1 week, 2 weeks, and 3 weeks). Each scatter plot compares the log2-transformed proteomic data between two specific time points, with the Pearson correlation coefficient (Corr) indicated in the top right corner of each plot. The histograms along the diagonal show the distribution of protein intensities at each time point. A high correlation coefficient close to 1 indicates a strong positive relationship between the datasets, suggesting consistent proteomic patterns across time. This consistent pattern reinforces the reliability of the proteomic alterations observed in response to ICV-STZ treatment in a time-dependent manner.

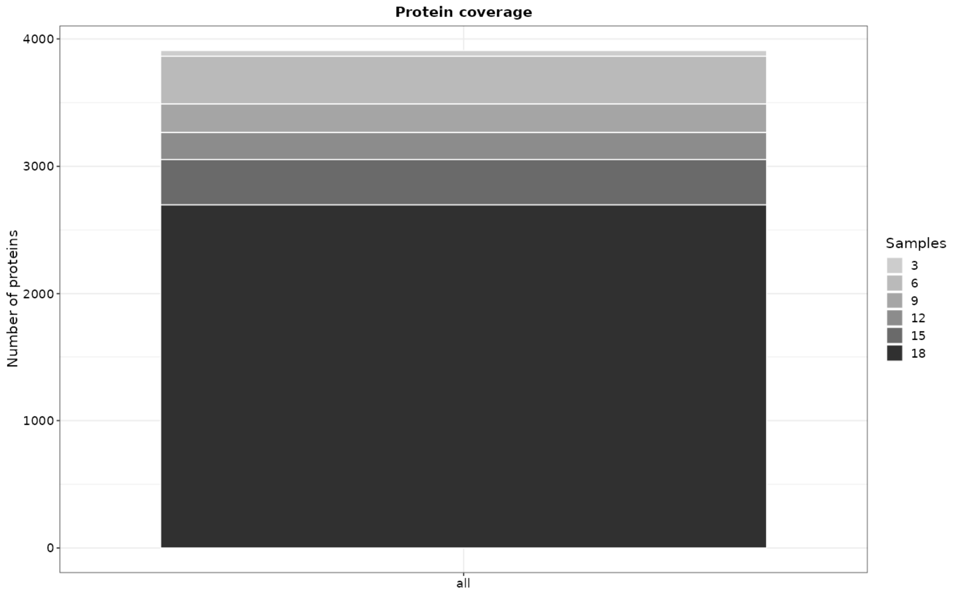

Figure S8: Comprehensive Protein Coverage in ICV-STZ Alzheimer's Disease Model. This figure demonstrates the overall protein coverage obtained from analyzing all samples in the ICV-STZ-treated Alzheimer's disease rodent model study. The stacked bar chart illustrates the cumulative number of proteins identified across all collected samples, with different shades of gray representing the incremental number of samples contributing to the total protein identification. Darker shades indicate the foundational number of proteins identified in fewer samples, while lighter shades represent additional proteins detected as the number of samples increases. The 'all' category on the x-axis denotes data aggregation from all time points and replicates. This visualization emphasizes the depth and comprehensiveness of the proteomic analysis conducted in this study, providing a global view of the protein landscape and the extent of proteomic coverage achieved.

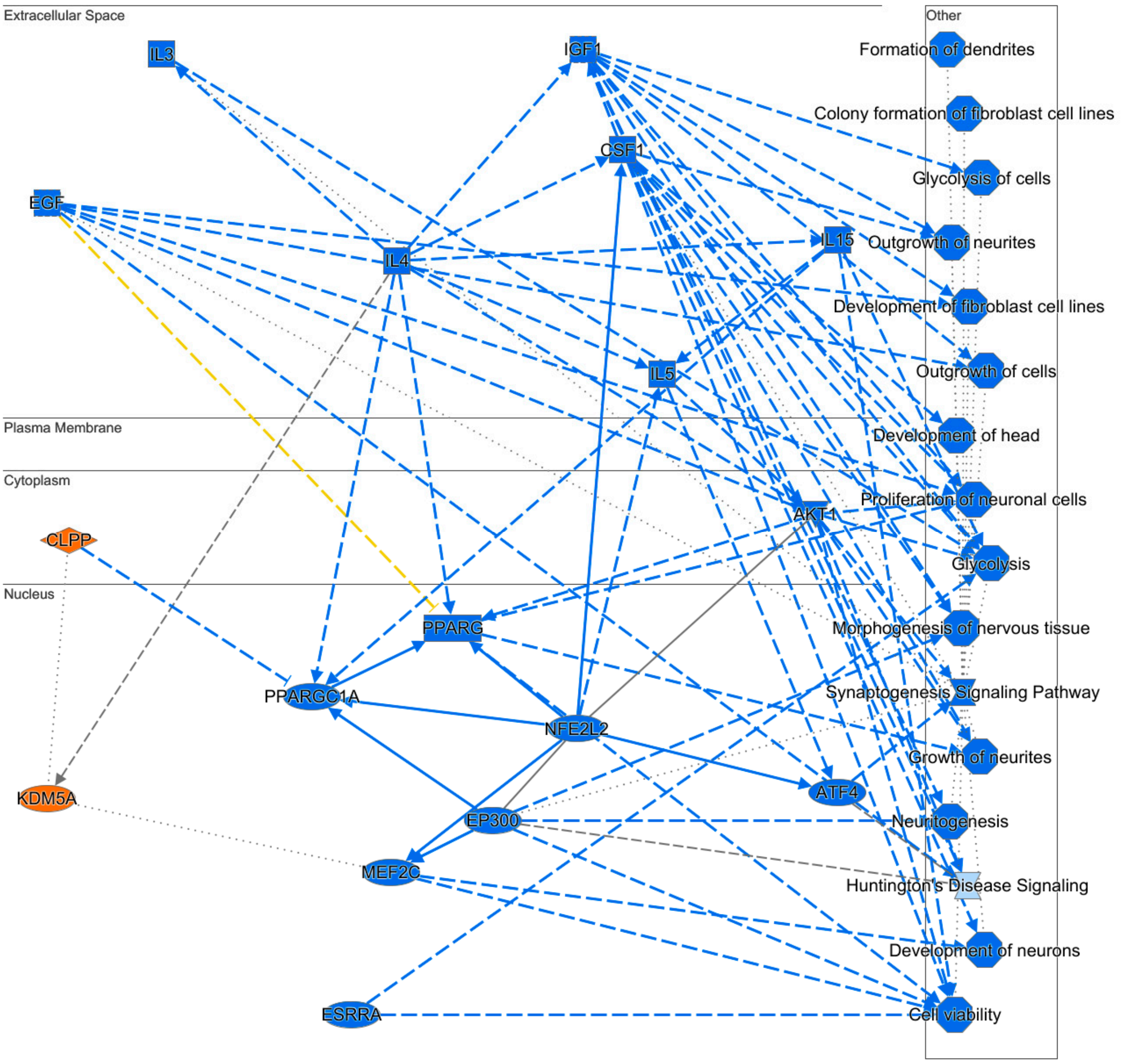

Figure S9: Comprehensive network of molecular interactions in an intracerebroventricular streptozotocin (ICV-STZ) treated Alzheimer's disease rodent model. Each node represents a distinct protein or gene, while the connecting lines illustrate the known molecular interactions based on IPA's extensive knowledge base. Direct interactions are marked with solid blue lines, suggesting a well-documented connection. Indirect interactions, indicative of multi-step biological processes, are shown with dashed gray lines. Highlighted nodes in orange pinpoint proteins with high connectivity, suggesting their significant roles in disease mechanisms. The directionality of the interactions is indicated by arrows; a line ending in an arrow denotes a promotional effect, whereas lines without arrowheads imply a non-directional association. This visual summary encapsulates the complex network of protein interactions, offering insights into potential targets for therapeutic intervention and biological pathways altered by Alzheimer's pathology. The figure was constructed by Ingenuity Pathway Analysis (IPA).
